# Statistical Use in Clinical Studies: Is There Evidence of a Methodological Shift?

**DOI:** 10.1371/journal.pone.0140159

**Published:** 2015-10-08

**Authors:** Dali Yi, Dihui Ma, Gaoming Li, Liang Zhou, Qin Xiao, Yanqi Zhang, Xiaoyu Liu, Hongru Chen, Julia Christine Pettigrew, Dong Yi, Ling Liu, Yazhou Wu

**Affiliations:** 1 Department of Health Statistics, College of Preventive Medicine, Third Military Medical University, Chongqing, China; 2 University of Washington, School of Arts and Sciences, Department of Biological Sciences, Seattle, Washington, United States of America; 3 University of Washington, School of Arts and Sciences, Department of Asian Language and Literature, Seattle, Washington, United States of America; University of Amsterdam, NETHERLANDS

## Abstract

**Background:**

Several studies indicate that the statistical education model and level in medical training fails to meet the demands of clinicians, especially when they want to understand published clinical research. We investigated how study designs and statistical methods in clinical studies have changed in the last twenty years, and we identified the current trends in study designs and statistical methods in clinical studies.

**Methods:**

We reviewed 838 eligible clinical study articles that were published in 1990, 2000, and 2010 in four journals New England Journal of Medicine, Lancet, Journal of the American Medical Association and Nature Medicine. The study types, study designs, sample designs, data quality controls, statistical methods and statistical software were examined.

**Results:**

Substantial changes occurred in the past twenty years. The majority of the studies focused on drug trials (61.6%, n = 516). In 1990, 2000, and 2010, there was an incremental increase in RCT studies (74.4%, 82.8%, and 84.0%, respectively, p = 0.013). Over time, there was increased attention on the details of selecting a sample and controlling bias, and there was a higher frequency of utilizing complex statistical methods. In 2010, the most common statistical methods were confidence interval for superiority and non-inferiority comparison (41.6%), survival analysis (28.5%), correction analysis for covariates (18.8%) and Logistic regression (15.3%).

**Conclusions:**

These findings indicate that statistical measures in clinical studies are continuously developing and that the credibility of clinical study results is increasing. These findings provide information for future changes in statistical training in medical education.

## Introduction

Recently, the design and statistical analysis of clinical studies have become increasingly strict and elaborate as a result of Evidence-based medicine (EBM). Many institutions published instructions for study design and statistical analysis of clinical studies, e.g. the guidelines for format and content of the clinical and statistical sections by Food and Drug Administration (FDA) 1988 [1–4]. Several clinical research articles have indicated a trend of increasingly sophisticated statistical techniques, and hidden information in the data can be shown more thoroughly and precisely with these techniques [5]. These techniques include methods to compare patterns (superiority, non-inferiority and equality) and data sets and the use of multiple comparison and survival analysis.

However, these improvements also make articles difficult to understand and grasp. A recent cross-sectional study found that less than half of the 277 sampled internal medicine residents had adequate statistical knowledge and understanding to follow the medical literature [6]. Several studies indicate that the statistical education model and level in present medical training fails to meet the demands of clinicians, especially when they want to understand published clinical research [7–10].

Medical training should include training in complex statistics [11], but there is uncertainty about what should be added and enhanced in the medical curriculum. Educators should agree on the type and depth of statistical knowledge that should be imparted on future clinicians.

Therefore, the object of this study was mainly to assess how study designs and statistical methods have changed in the last twenty years and to determine the current trends in study design and statistical methods in clinical studies.

## Methods

### Inclusion criteria

There are two main types of clinical studies: clinical trials (also called interventional studies) and observational studies (PubMed homepage, ClinicalTrials.gov). So in this study, the inclusion criteria can be defined as following:

The type of study: the clinical trials and observational studies;

Participants (articles): The articles from New England Journal of Medicine (NEJM), Lancet, Journal of the American Medical Association (JAMA) and Nature Medicine;

Intervention (treatment factors or exposure factors): Observational study (descriptive study, case-control study and cohort study), drug trial, medical apparatus and instruments, operation methods, health education, diet therapy, exercise therapy, stem cell therapy, et al;

Control: Exposure factors (observational study), other interventions or placebo(clinical trials);

Outcome: the statistical methods of inclusion articles, such as descriptive statistics, t-test, ANOVA, Survival analysis, and statistical software et al.

### Exclusion criteria

Comments, case reports, systematic reviews, meta-analyses, genome-wide analyses and articles did not involve primary or secondary data analysis were excluded from the study. The articles were also excluded from the study if the sample size was less than 10.

### Selected Articles

**Fig 1:**
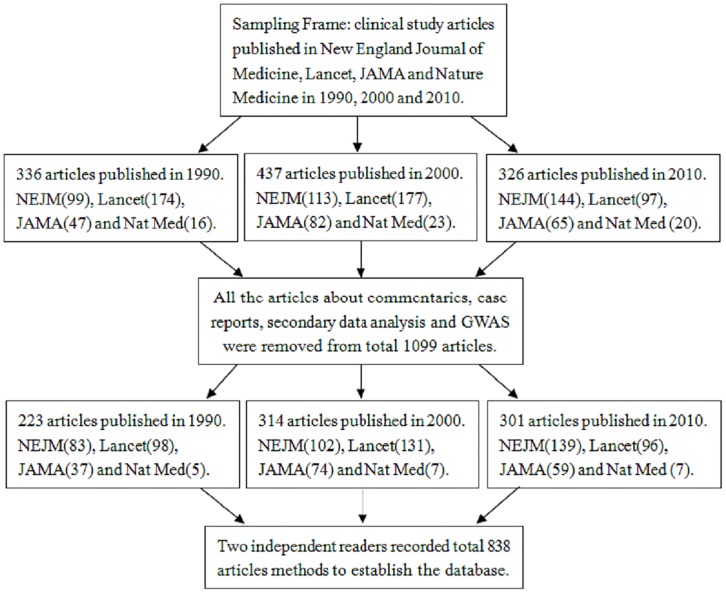
Flow chart of the selection criteria for the content analysis.

To assess how statistical methodology of clinical studies has changed in the last twenty years. The articles were sampled in New England Journal of Medicine (NEJM), Lancet, Journal of the American Medical Association (JAMA) and Nature Medicine on three time points 1990, 2000, and 2010 (Fig 1).

The sampling frame for articles included all issues of the selected four journals in the years 1990, 2000 and 2010 and were web-searched only on PubMed homepage. On PubMed homepage, regarding article types, the type of clinical studies was selected and then entered the site of ClinicalTrials.gov. ClinicalTrials.gov is a registry and results database of publicly and privately supported clinical studies (clinical trials and observational studies) of human participants conducted around the world. Then the "PubMed Advanced Search Builder" can be obtained, for example, the search builder of clinical trials of Lancet in 1990 is as: Search ("Lancet (London, England)"[Journal]) AND ("1990"[Date—Publication]: "1990"[Date—Publication]) Filters: Clinical Trial. In this way, all articles of clinical studies published in the selected four journals and on three years can be searched to conduct this content analysis.

All articles within those issues which load down from website of ClinicalTrials.gov were then evaluated for eligibility according to inclusion/exclusion criteria. Eligible articles were those in which authors implemented a study and analyzed primary or secondary data for the clinical trials and observational studies. Specifically, the articles of original clinical trial and clinical investigation were eligible for inclusion, regarding to RCTs, case-control studies, cohort studies and descriptive studies. As commentary, case reports, systematic reviews, meta-analyses, genome-wide analyses, and articles did not involve primary or secondary data analysis, they were excluded from the study.

### Data collection

A data collection schedule was discussed within the research group. The main contents of the discussion include: which aspects can reflect the statistical methodological shift of clinical studies, what categorizations should be included in each aspect (as determined by Table of Contents)? In this study, the aspects of statistical trends should be: study types, study designs, sample designs, data quality control, statistical methods and statistical software.

There are some different ideas for categorizations of statistical methods. In this study, the pre-determined categorization for statistical methods was done similarly to what Arnold LD et al. did previously [11]. The statistical methods were not specified in some articles clearly. For example, if the authors calculated hazard ratios but did not specify the type of survival analysis, the articles were coded as "Survival analysis". If no specific correction analysis was mentioned but the word "adjusted" was used, the article was coded as using correction analysis for covariates.

Two readers with masters-level training in biostatistics independently abstracted data pertaining to study types, study designs, sample designs, data quality control, statistical methods and statistical software. After abstracting the data for each article, two readers entered data into independent files and then merged the entries into one file for data reconciliation by Epidata 3.0.

Except input error, instances of discordant information were flagged (less than 10% in 838). Two readers reconciled the data case-by-case referencing the article, when discrepancies were present e.g. the number of used statistical methods is different for one article between two readers. When discrepancies could not be resolved by referencing the article, the readers would only consult statisticians (corresponding authors) until they reached an agreement.

### Data analysis

Descriptive statistics were generated for each data category (e.g. the number of statistical designs/methods), overall by year of publication. Significant differences for variables (e.g. prevalence of statistical designs/methods) over the three study years (1990, 2000, and 2010) were examined using chi-square and Fisher exact test, and p-values of less than 0.05 were considered to be statistically significant. The software SPSS v.18 and Epidata 3.0 were used for all analyses.

## Results

### Study types

After searching in PubMed, 1,099 clinical study articles were got in four journals. Excluding the 261 articles, which were adapting to the exclusion criteria, a total of 838 eligible articles were included, including 223 (26.6%) from 1990, 314 (37.5%) from 2000, and 301 (35.9%) from 2010. As shown in Table 1, the majority of the studies focused on drug trials (61.6%, n = 516). There were significant differences in three study types over the three years, including drug trial (p = 0.004), operation method (p = 0.028) and other types (p = 0.008). The so-called "other types" include health Education, diet therapy, exercise therapy, stem cell therapy, etc. There was no significant difference in study type of medical apparatus and instruments over the three years (3.1% in 1990, 6.1% in 2000, and 3.0% in 2010; p = 0.110).

**Table 1:** Study types of 838 articles published in 1990, 2000, and 2010.

Categories	Total	Article Year 1990(n = 223)	Article Year 2000(n = 314)	Article Year 2010(n = 301)	*χ* ^2^	*P* *
Drug trial	516(61.6%)	157(70.4%)	190(60.5%)	169(56.1%)	11.246	0.004
Medical apparatus and instruments	35(4.2%)	7(3.1%)	19(6.1%)	9(3.0%)	4.415	0.110
Operation method	51(6.1%)	6(2.7%)	26(8.3%)	19(6.3%)	7.170	0.028
Other types^△^	236(28.1%)	53(23.8%)	79(25.1%)	104(34.6%)	9.603	0.008

*Chi-square for difference by year,

^△^including: Health Education, Diet therapy, Exercise therapy, Stem cell therapy etc.

### Study designs

As demonstrated in Table 2, the most common clinical study design was the randomized controlled trial (RCT) (81.1%, n = 679). The number of descriptive studies decreased (12.2% in 1990, 7.6% in 2000, and 5.3% in 2010; p = 0.017). The number of case-control studies decreased (11.2% in 1990, 5.1% in 2000, and 0.7% in 2010; p<0.001). The number of cohort studies increased (2.2% in 1990, 4.5% in 2000, and 10.0% in 2010; p<0.001). The number of published RCTs increased (74.4% in 1990, 82.8% in 2000, and 84.0% in 2010; p = 0.013).

**Table 2:** Study designs of 838 articles published in 1990, 2000, and 2010.

	Total	Article Year 1990(n = 223)	Article Year 2000(n = 314)	Article Year 2010(n = 301)	*χ* ^2^	*P* *
Clinical Design						
Descriptive^#^	67(8.0%)	27(12.2%)	24(7.6%)	16(5.3%)	8.118	0.017
Case-control	43(5.1%)	25(11.2%)	16(5.1%)	2(0.7%)	29.269	<0.001
Cohort	49(5.8%)	5(2.2%)	14(4.5%)	30(10.0%)	15.643	<0.001
RCT	679(81.1%)	166(74.4%)	260(82.8%)	253(84.0%)	8.732	0.013
Control Design						
Parallel	687(82.0%)	170(76.2%)	261(83.1%)	256(85.0%)	7.183	0.028
Crossover	35(4.2%)	17(7.6%)	13(4.1%)	5(1.7%)	11.379	0.003
Factorial	28(3.3%)	7(3.2%)	7(2.2%)	14(4.7%)	2.830	0.243
Sequential	10(1.2%)	4(1.8%)	3(1.0%)	3(1.0%)	0.931	0.628
Other	78(9.3%)	25(11.2%)	30(9.6%)	23(7.6%)	1.970	0.374
Comparison Design						
Difference	463(55.3%)	167(74.9%)	154(49.0%)	142(47.2%)	47.610	<0.001
Superiority	210(25.1%)	33(14.8%)	85(27.1%)	92(30.6%)	18.037	<0.001
Non-inferiority	138(16.5%)	18(8.1%)	65(20.7%)	55(18.3%)	16.230	<0.001
Equality	27(3.2%)	5(2.2%)	10(3.2%)	12(4.0%)	1.253	0.535
Primary endpoint						
	675(80.6%)	129(57.8%)	264(84.1%)	282(93.7%)	109.009	<0.001

^**#**^ Descriptive: cross-sectional study, sampling survey and prevalence study;

*Chi-square test for differences among years.

The majority of the control design were parallel (82.0%, n = 687). There were no significant differences in three control designs over the three years, including factorial (p = 0.243), sequential (p = 0.628) and other controls (p = 0.374). The number of parallel controls increased (76.2% in 1990, 83.1% in 2000, and 85.0% in 2010; p = 0.028). The number of crossover controls decreased (7.6% in 1990, 4.1% in 2000, and 1.7% in 2010; p = 0.003).

The majority of the comparison designs focused on difference test (55.3%, n = 463). The number of difference articles increased (57.8% in 1990, 84.1% in 2000, and 93.7% in 2010; p<0.001). The number of superiority studies increased (14.8% in 1990, 27.1% in 2000, and 30.6% in 2010; p<0.001). And the number of non-inferiority studies increased (57.8% in 1990, 84.1% in 2000, and 93.7% in 2010; p<0.001). The number of studies using primary endpoints also increased (57.8% in 1990, 84.1% in 2000, and 93.7% in 2010; p<0.001).

### Sample designs

As shown in Table 3, the number of studies that used multiple centers increased (26.9% in 1990, 63.7% in 2000, and 81.4% in 2010; p<0.001). More studies reported the use of two groups or three or more groups (89.2% in 1990, 92.4% in 2000, and 96.3% in 2010; p = 0.006). Over the three years, there was a significant increase in the reporting of sample estimation methods (21.5% in 1990, 48.4% in 2000, and 79.4% in 2010; p<0.001) and power estimation (17.0% in 1990, 45.5% in 2000, and 77.1% in 2010; p<0.001).

**Table 3:** Sample designs of 838 articles published in 1990, 2000, and 2010.

	Total	Article Year 1990(n = 223)	Article Year 2000(n = 314)	Article Year 2010(n = 301)	*χ* ^2^	*P* *
Sample source						
Single center	333(39.7%)	163(73.1%)	114(36.3%)	56(18.6%)	161.294	<0.001
Multiple centers	505(60.3%)	60(26.9%)	200(63.7%)	245(81.4%)	161.294	<0.001
Sample groups						
One group	59(7.0%)	24(10.8%)	24(7.6%)	11(3.7%)	10.167	0.006
Two groups	577(68.9%)	147(65.9%)	209(66.6%)	221(73.4%)	4.594	0.101
Three or more groups	202(24.1%)	52(23.3%)	81(25.8%)	69(22.9%)	0.796	0.672
Sample estimation method						
	439(52.4%)	48(21.5%)	152(48.4%)	239(79.4%)	175.219	<0.001
Power estimation						
	413(49.3%)	38(17.0%)	143(45.5%)	232(77.1%)	187.534	<0.001

*Chi-square test for differences among years.

### Data quality controls

Four indexes of data quality are shown in Table 4. The first clinical trial register (CTR) was established in February 2002 by the American National Institutes of Health (NIH), National Library of Medicine (NLM) and FDA [12], so clinical study articles published in 1990 and 2000 did not register on the CTR. However, in 2010, the proportion of registered studies was 58.1%.

**Table 4:** Data quality control of 838 articles published in 1990, 2000, and 2010.

	Total	Article Year 1990(n = 223)	Article Year 2000(n = 314)	Article Year 2010(n = 301)	*χ* ^2^	*P* *
Trial registration						
	175(20.9%)	0(0.0%)	0(0.0%)	175(58.1%)	—	—
Blindness						
Open	78(9.3%)	22(9.9%)	22(7.0%)	34(11.3%)	3.461	0.177
Single-blind	325(38.8%)	75(33.6%)	130(41.4%)	120(39.9%)	3.548	0.170
Double-blind	429(51.9%)	126(56.5%)	162(51.6%)	147(48.8%)	3.035	0.219
Data entry						
Single entry	538(64.2%)	185(83.0%)	161(51.3%)	192(63.8%)	56.995	<0.001
Double entry	106(12.6%)	5(2.2%)	11(3.5%)	90(29.9%)	126.703	<0.001
No mention	194(23.2%)	33(14.8%)	142(45.2%)	19(6.3%)	142.699	<0.001
FAS-PPS-SS^#^						
	476(56.8%)	31(13.9%)	215(68.5%)	230(76.4%)	231.864	<0.001

*Chi-square test for differences by years.

^#^FAS- full analysis set, PPS -per-protocol set, SS-safety analysis set.

Though there was no significant difference in the use of blindness over the three years (p = 0.117 for open, p = 0.170 for single-blind, p = 0.219 for double-blind), but there were significant differences in the form of data entry over the three years (all p<0.001). An increasing number of studies reported on Data Sets (DS) (13.9% in 1990, 68.5% in 2000, and 76.4% in 2010; P<0.001).

### Statistical methods

As demonstrated in Table 5, the most commonly reported statistics in the reviewed articles were descriptive statistics (100.0%), ANOVA (47.2%) and T-test (36.3%). Between 1990 and 2010, there was no significant difference in the following statistics: including descriptive statistics, chi-square, fisher exact, Mantel-Haenszel, T-test and ANOVA (p>0.05).

**Table 5:** Statistical methods published in 1990, 2000, and 2010^△^.

	Total	Article Year 1990(n = 223)	Article Year 2000(n = 314)	Article Year 2010(n = 301)	*χ* ^2^	*P* *
Descriptive statistics^#^	838(100.0%)	223(100.0%)	314(100.0%)	301(100.0%)	—	—
Chi-square	275(32.8%)	83(37.1%)	97(30.9%)	95(31.6%)	5.236	0.062
Fisher exact	92(11.0%)	22(10.0%)	38(12.0%)	32(10.6%)	1.707	0.426
Mantel-Haenszel	80(9.5%)	4(2.0%)	26(8.3%)	49(16.3%)	2.252	0.260
T-test	304(36.3%)	78(35.0%)	114(36.3%)	112(37.2%)	1.345	0.520
ANOVA^★^	394(47.0%)	110(49.3%)	148(47.1%)	136(45.2%)	5.636	0.060
Interim analysis	53(6.3%)	6(2.5%)	19(6.2%)	28(9.3%)	9.515	0.009
Correlation analysis	226(27.0%)	40(17.9%)	75(23.9%)	111(36.9%)	25.755	<0.001
Simple linear regression	196(23.4%)	35(15.7%)	65(20.7%)	96(31.9%)	20.784	<0.001
Multiple comparison	63(7.5%)	12(5.6%)	20(6.3%)	31(10.2%)	5.409	0.047
Non-parametric test	235(28.0%)	52(23.1%)	104(33.2%)	79(26.2%)	6.961	0.031
Wilcoxon test	117(14.0%)	27(12.3%)	43(13.6%)	47(15.6%)	1.341	0.511
Logistic regression	128(15.3%)	27(12.3%)	49(15.6%)	52(17.3%)	7.686	0.021
Survival analysis	239(28.5%)	34(15.3%)	74(23.6%)	131(43.4%)	56.279	<0.001
Cox models	134(16.0%)	17(7.7%)	43(13.6%)	74(24.6%)	29.404	<0.001
Kaplan Meier	117(13.9%)	8(3.6%)	37(11.7%)	72(23.9%)	46.070	<0.001
Sensitivity analysis	29(3.5%)	3(1.3%)	10(3.3%)	16(5.3%)	6.159	0.046
Transformation	103(12.3%)	16(7.2%)	28(8.8%)	59(19.6%)	23.651	<0.001
Correction analysis for covariates	158(18.8%)	19(8.3%)	64(20.3%)	76(25.1%)	23.966	<0.001
Confidence Interval						
Difference	249(29.7%)	101(45.3%)	84(26.8%)	64(21.3%)	37.524	<0.001
Superiority	210(25.1%)	33(14.8%)	85(27.1%)	92(30.6%)	18.037	<0.001
Non-inferiority	138(16.5%)	18(8.1%)	65(20.7%)	55(18.3%)	16.230	<0.001
Equality	27(3.2%)	5(2.2%)	10(3.2%)	12(4.0%)	1.253	0.242

*Chi-square test for differences among years.

^△^Excludes statistics in which there were n<15 across all three years of review.

^#^Including: means, standard variation, median, percentages.etc.

^★^Including repeated measurement analysis.

From 1990 to 2010, there was a increase in the following statistics: specifically logistic regression (12.3% in 1990, 15.6% in 2000, and 17.3% in 2010; p = 0.021), multiple comparison (5.6% in 1990, 6.3% in 2000, and 10.2% in 2010; p = 0.047), Cox models (7.7% in 1990, 13.6% in 2000, and 24.6% in 2010; p = 0.031), Kaplan Meier tests (3.6% in 1990, 11.7% in 2000, and 23.9% in 2010; p = 0.031), sensitivity analysis (1.3% in 1990, 3.3% in 2000, and 5.3% in 2010; p = 0.046) and correction analysis for covariates (8.3% in 1990, 20.3% in 2000, and 25.1% in 2010; p<0.001).

From 1990 to 2010, there was a significant increase in the reporting of confidence interval, specifically superiority (14.8% in 1990, 27.1% in 2000, and 30.6% in 2010; p<0.001). From 1990 to 2010, there was a significant difference in the reporting of non-inferiority (8.1% in 1990, 20.7% in 2000, and 18.3% in 2010; p<0.001). But there was a significant decrease in the reporting of difference (45.3% in 1990, 26.8% in 2000, and 21.3% in 2010; p<0.001).

Interim analysis was reported infrequently overall, with significantly differences over time (2.5% in 1990, 6.2% in 2000, and 9.3% in 2010; p = 0.009).

### Statistical software

As recorded in Table 6, there was a significant increase over time in reporting of SAS (13.5% in 1990, 41.7% in 2000, and 46.8% in 2010; p<0.001) and STATA (3.1% in 1990, 11.5% in 2000, and 10.6% in 2010; p = 0.002). There was no significant difference over time in reporting of SPSS (p = 0.104) and R software (p = 0.082).

**Table 6:** Statistical software published in 1990, 2000, and 2010.

	Total	Article Year 1990(n = 223)	Article Year 2000(n = 314)	Article Year 2010(n = 301)	*χ* ^2^	*P* *
Statistical software						
SPSS	256(30.5%)	56(25.1%)	99(31.5%)	101(33.6%)	4.531	0.104
SAS	302(36.0%)	30(13.5%)	131(41.7%)	141(46.8%)	69.993	<0.001
STATA	75(8.9%)	7(3.1%)	36(11.5%)	32(10.6%)	12.722	0.002
R	30(3.6%)	3(1.3%)	12(3.8%)	15(5.0%)	4.997	0.082
Other software	75(8.9%)	39(17.5%)	26(8.3%)	10(3.3%)	31.824	<0.001
Not mention	100(11.9%)	88(39.5%)	10(3.2%)	2(0.7%)	220.046	<0.001
Data Management						
Database^#^	388(46.3%)	47(21.1%)	132(42.0%)	209(69.4%)	124.156	<0.001
Excel^※^	162(19.3%)	55(24.7%)	82(26.1%)	25(8.3%)	36.795	<0.001
Not mention	288(34.4%)	121(54.3%)	100(31.8%)	67(22.3%)	59.571	<0.001

*Chi-square test for differences on three years.

^#^Database includes EpiData, Oracle, Access and Others. If the article mentioned the data is input into database, then it was divided into "Others".

^※^Excel is not a database.

The number of studies that use database to manage data increased (21.1% in 1990, 42.0% in 2000, and 69.4% in 2010; p<0.001).

## Discussion

The choice of these four general medicine journals for this study is strength, as they should be the leading medical journals with an extremely broad readership. They are widely read by clinicians in a variety of specialties and publish across a range of clinically related issues, so they are certainly representative of published paper in general. For the generalizability of findings, which journals could be included in have been discussed with PLOS ONE Academic Editors for many times.

Although a large number of 838 eligible articles were included in this study, the focus on these four general medicine journals for this content analysis is a limitation as it restricts generalizability of findings and does not account for variation by specialty. For example, preferred choice of study designs and data analysis expectations in surgical fields may differ from those in psychiatry or pediatrics. Thus, trends in study design and analytic techniques present here may differ from journals with more directed target audiences and area of focus. To assess differences in the use of statistical methods in general medicine journals and specialized journals, we identified reviews of statistical methods used in specialized journals. A 1995 study comparing prevalence and use of statistical analysis found that rheumatology journals [13] tended to use fewer and simpler statistics than general medicine journals. So this study still have important guidance for statistics education. Meanwhile, if this content analysis was extended to include articles from other integrative journals, then it is anticipated that individual findings would vary but that overall trends of increasing statistical complexity over the decades would be similar.

In this content analysis and individual findings, what are overall trends of increasing statistical complexity over the decades? Regarding trends of study types, drug trials decreased over time, but other types (e.g. some new skills of health education, diet therapy, exercise therapy, stem cell therapy, etc.) occurred with more frequency. Regarding trends of study design, descriptive and case-control studies occurred with less frequency over time; Cohort and RCT studies occurred with more frequency. this phenomenon suggests that study design has become increasingly rigorous in the last twenty years. Meanwhile, the tendency to use statistical hypothesis testing may be associated with a decrease in studies that compared difference and an increase in studies that utilized superiority and non-inferiority, especially in 2010; this phenomenon suggests that statistical hypothesis testing has become more accurate than before.

Regarding trends of sample design and data quality control, they are two key aspects of clinical study results, as appropriate sample design and rigorous data quality control can improve the reliability and credibility of clinical study results [14–17]. The results of the present study also show that the use of multiple centers, sample estimation methods, power estimation and data set comparisons has increased over time. Some of these methods, such as sample estimation methods, power estimation and FAS-PPS-SS, were used in earlier studies but were less likely to be included in studies published before clinical trial guidelines were published by the International Conference on Harmonization (ICH-E9) in 1998 [4]. These trends show that the EBM and journals have been increasingly strict on the quality of trials.

Regarding trends of statistical methods, the proportion of papers that reported using multiple comparison, survival analysis (Cox models and Kaplan-Meier), sensitivity analysis, interim analysis, confidence interval (superiority and non-inferiority) and correction analysis increased significantly from 1990 to 2010. These complex statistical methods require strong statistical understanding to interpret their application and the results. Some of these technologies were less likely to be included before the statistical analysis guidelines for clinical trials were published in 1992 and 1993 [2,3]. Because the rules of statistical analysis guidelines have clearly specified many complex statistical methods must be concluded in, e.g. confidence interval (superiority and non-inferiority). The phenomenon that complex statistical methods are used more frequently indicates that journals are more strict regarding the accuracy and type of statistical analyses that are reported in articles [7,11,18].

Only 0.7% of the surveyed articles didn’t mention the type of statistical software that was used for the analyses in 2010, and nearly 90.0% of those articles used SAS, SPSS, STATA and R. The data on statistical software show that professional statistical software is used with increasing frequency, and journal editors demand more precise details of statistical methods.

From 1990 to 2010, we note that there has been little change in content of medical education [9]. Even in instances where statistical content of training may have been revised and updated, the degree to which material is covered may be limited, e.g. confidence interval (Superiority and non-inferiority), sensitivity analysis, interim analysis, and correction analysis even not covered in most textbook. This contrasts with the substantial increases in frequency and complexity of statistical reporting.

While our findings do not directly suggest that medical education necessarily needs to be modified, the statistical reporting trends described may have implications for medical education. Similarly, while this study does not provide data to suggest that improved statistical knowledge could translate to more effective use of the literature, we do propose that physicians’ familiarity with certain (complex) statistical approaches may assist them in critically evaluating and weighing the literature.

To this end, medical educators may wish to be aware of the benefits and limitations of different and more complex statistical strategies as they try to teach certain topical content or critical evaluation skills. Moreover, as future and current clinicians engage in a life-long learning process, findings from this study may be used as part of the discussion about statistical training across the continuum of medical education.

## Supporting Information

S1 PRISMA Checklist PRISMA 2009 Checklist. (DOC)
