# Supplementary material for: Statistical Use in Clinical Studies: Is There Evidence of a Methodological Shift?
Source: PLoS One. 2015 Oct 8;10(10):e0140159. doi: 10.1371/journal.pone.0140159 (PMC4598157; doi:10.1371/journal.pone.0140159)
Supplement: S1 PRISMA Checklist — (DOC) [file pone.0140159.s001.doc]

PRISMA 2009 Checklist

| **Section/topic** | **#** | **Checklist item** | **Reported on page #** |
| --- | --- | --- | --- |
| TITLE | | | |
| Title | 1 | Statistical Use in Clinical Trials: Is There Evidence of a Methodological Shift? | Page 1. |
| ABSTRACT | | | |
| Structured summary | 2 | Background: Several studies indicate that the statistical education model and level in medical training fails to meet the demands of clinicians, especially when they want to understand published clinical research. We investigated how study designs and statistical methods in clinical studies have changed in the last twenty years, and we identified the current trends in study designs and statistical methods in clinical studies.  Methods: We reviewed 838 clinical study articles that were published in 1990, 2000, and 2010 in four journals New England Journal of Medicine, Lancet, Journal of the American Medical Association and Nature Medicine. The study types, study designs, sample designs, data quality controls, statistical methods and statistical software were examined.  Results: Substantial changes occurred in the past twenty years. The majority of the studies focused on drug trials (61.6%, n= 516). In 1990, 2000, and 2010, there was an incremental increase in RCT studies (74.4%, 82.8%, and 84.0%, respectively, p=0.013). Over time, there was increased attention on the details of selecting a sample and controlling bias, and there was a higher frequency of utilizing advanced statistical methods. In 2010, the most common advanced statistical methods were confidence interval for superiority and non-inferiority comparison (41.6%), survival analysis (28.5%), correction analysis for covariates (18.8%) and Logistic regression (15.3%).  Conclusions: These findings indicate that statistical measures in clinical studies are continuously developing and that the credibility of clinical study results is increasing. These findings provide information for future changes in statistical training in medical education. | Page 1-2. |
| INTRODUCTION | | | |
| Rationale | 3 | We reviewed 838 clinical study articles that were published in 1990, 2000, and 2010 in four journals New England Journal of Medicine, Lancet, Journal of the American Medical Association and Nature Medicine. The study types, study designs, sample designs, data quality controls, statistical methods and statistical software were examined. | Page 3-4. |
| Objectives | 4 | The object of this study was mainly to assess how study designs and statistical methods have changed in the last twenty years and to determine the current trends in study design and statistical methods in clinical studies. | Page 2. |
| METHODS | | | |
| Protocol and registration | 5 | N/A. | N/A. |
| Eligibility criteria | 6 | All clinical study articles are searched by keyword "clinical trials" on PubMed homepage within selected four journals. In PubMed, a "clinical trials" (actually clinical studies) involves researches using human volunteers (also called participants) that is intended to add to medical knowledge. There are two main types of clinical studies: clinical trials (also called interventional studies) and observational studies.  All clinical study articles were then evaluated for eligibility. Eligible articles were those in which the authors implemented a study and analyzed primary data. Specifically, the following categories of articles were eligible for inclusion: original contribution, clinical investigation. So the study designs include: randomised designs, non-randomised designs, quasi-experimental or observational designs.  Commentaries, case reports, [systematic](app:ds:systematic) [review](app:ds:review)s, meta-analyses and genome-wide analyses were excluded from the study. The articles were also excluded from the study if the sample size was less than 10. | Page 3. |
| Information sources | 7 | The samples of papers are from four journals, including New England Journal of Medicine (NEJM), Lancet, Journal of the American Medical Association (JAMA) and Nature Medicine. The choice of these journals for this study is strength, as they are leading medical journals with an extremely broad readership (probably the top 5%). They are widely read by clinicians in a variety of specialties and publish across a range of clinically related issues. Which journals could be included in have been discussed with PLOS ONE Academic Editors for many times.  To assess how study designs and statistical methods have changed in the last twenty years, the articles are sampled on three time points 1990, 2000, and 2010. And the sampling frame included all clinical research articles in the four journals on 1990, 2000, and 2010. | Page 3. |
| Search | 8 | All clinical study articles are searched by keyword "clinical trials" on PubMed homepage within selected four journals. In PubMed, a "clinical trials" (actually clinical studies) involves researches using human volunteers (also called participants) that is intended to add to medical knowledge. There are two main types of clinical studies: clinical trials (also called interventional studies) and observational studies. | Page 3. |
| Study selection | 9 | All clinical study articles were then evaluated for eligibility. Eligible articles were those in which the authors implemented a study and analyzed primary data. Specifically, the following categories of articles were eligible for inclusion: original contribution, clinical investigation. So the study designs include: randomised designs, non-randomised designs, quasi-experimental or observational designs.  Commentaries, case reports, [systematic](app:ds:systematic) [review](app:ds:review)s, meta-analyses and genome-wide analyses were excluded from the study. The articles were also excluded from the study if the sample size was less than 10. | Page 3. |
| Data collection process | 10 | Two readers independently abstracted data pertaining to study types (table 1), study designs (table 2), sample designs (table 3), data quality control (table 4), statistical methods (table 5) and statistical software (table 6). When data did not clearly fall into one of the pre-determined categories (e.g., the study design was not clearly specified), coding was discussed and a consensus was reached.  There are some different ideas for categorizations of statistical methods. In this study, the categorization of table 5 for statistical methods was mainly referred to. If the authors calculated hazard ratios but did not specify the type of survival analysis, the articles were coded as "Survival analysis". If no specific correction analysis was mentioned but the word "adjusted" was used, the article was coded as using correction analysis for covariates. Excludes statistics in which there were n＜15 across all three years of review (table 5). | Page 3-4. |
| Data items | 11 | Two readers read the articles and recorded the statistical measures and procedures in each article, and the two entries were merged into one file for data reconciliation. Instances of discordant information were flagged, and the readers reconciled the data case-by-case, referencing the article when discrepancies were present. When discrepancies could not be resolved by referencing the article, the readers would only consult statisticians (corresponding authors) until they reached an agreement. | Page 4. |
| Risk of bias in individual studies | 12 | N/A. | N/A. |
| Summary measures | 13 | Descriptive statistics were generated for each data category and were used to describe data for all years and by year of publication. Significant differences for variables over the three study years (1990, 2000, and 2010) were examined using chi-square and Fisher exact test, and p-values of less than 0.05 were considered to be statistically significant. The software SPSS v.18 was used for all analyses. | Page 4. |
| Synthesis of results | 14 | Study types, study designs, sample designs, data quality controls, statistical methods, and statistical software. | Page 5-7. |
| Risk of bias across studies | 15 | N/A. | N/A. |
| Additional analyses | 16 | N/A. | N/A. |
| RESULTS | | | |
| Study selection | 17 | To conduct this content analysis, we included 1,099 clinical study articles in four journals. A total of 838 eligible articles were reviewed, including 223 (26.6%) from 1990, 314 (37.5%) from 2000, and 301 (35.9%) from 2010. | Page 5. |
| Study characteristics | 18 | In PubMed, a "clinical trials" (actually clinical studies) involves researches using human volunteers (also called participants) that is intended to add to medical knowledge. There are two main types of clinical studies: clinical trials (also called interventional studies) and observational studies. | Page 3. |
| Risk of bias within studies | 19 | N/A. | N/A. |
| Results of individual studies | 20 | N/A. | N/A. |
| Synthesis of results | 21 | Study types, study designs, sample designs, data quality controls, statistical methods, and statistical software. | Page 5-7. |
| Risk of bias across study | 22 | N/A. | N/A. |
| Additional analysis | 23 | N/A. | N/A. |
| DISCUSSION | | | |
| Summary of evidence | 24 | In this study, the samples of papers are from four journals. They are certainly representative of published paper in general. a large number of 838 eligible articles were reviewed by two readers. And valuable information can be gleaned from the six tables in the present study. Regarding study types, drug trials increased over time, some new skills of other types (e.g. health education, diet therapy, exercise therapy, stem cell therapy, etc.) occurred with more frequency. | Page 8. |
| Limitations | 25 | N/A. | N/A. |
| Conclusions | 26 | These findings indicate that statistical measures in clinical studies are continuously developing and that the credibility of clinical study results is increasing. These findings provide information for future changes in statistical training in medical education. | Page 2. |
| FUNDING | | | |
| Funding | 27 | This work was partially supported by the National Natural Science Foundation of China (No.81273178, No. 81172773), Research Projects of Postgraduate Education and Teaching Reform in Chongqing, China (No.yjg123101) and Higher Education Scientific Research Subject of the Higher Education Institute from 2013 to 2014 in Chongqing (No.CQGJ13C652). | Page 9. |
